# Norwegian scabies - rare case of atypical manifestation*

**DOI:** 10.1590/abd1806-4841.20164811

**Published:** 2016

**Authors:** Karina Corrêa Ebrahim, Júlia Barazetti Alves, Lísias de Araújo Tomé, Carlos Floriano de Moraes, Arianne Ditzel Gaspar, Karin Fernanda Franck, Mohamad Ali Hussein, Lucas Raiser da Cruz, Leonardo Duque Ebrahim, Luis Felipe de Oliveira Sidney

**Affiliations:** 1 Centro Universitário da Fundação Assis Gurgacz (FAG) – Cascavel (PR), Brazil; 2 Unidade de Pronto Atendimento Veneza (UPA) – Cascavel (PR), Brazil; 3 Private clinic - Cascavel (PR), Brazil; 4 Hospital do Câncer de Cascavel (Uopeccan) – Cascavel (PR), Brazil

**Keywords:** Mite infestation, Parasitic diseases, Scabies

## Abstract

Human scabies affects all social classes and different races around the world. It is highly contagious, but the exact figures on its prevalence are unknown. A 19-year-old male patient was admitted to the emergency room reporting fever (38°C) and multiple lesions throughout the body, except face, soles, and palms. Lesions were non-pruritic, which hampered the initial diagnostic suspicion. Skin biopsy was performed, and the final diagnosis was crusted scabies (Norwegian). It was concluded that human scabies is a significant epidemic disease, due to its different clinical manifestations, and because it is extremely contagious.

## INTRODUCTION

Human scabies is an infestation caused by female mites (*Sarcoptes scabiei* var. *hominis*), which lay 40-50 eggs in their 4-6 weeks of life. It is highly contagious and affects all social classes and different races around the world. However, the exact figures on its prevalence is unknown.^1^ It is also called Norwegian scabies because it was described in Norway by Danielssen and Boeck as a type of scabies infestation cause by millions of mites in patients with leprosy. The authors studied cases of scabies in patients with sensory paresthesias, cognitive impairment, physical disability, and immunosuppression.^2^

Patients develop severe erythroderma in over 90% of their body surface, followed by intense erythema and desquamation. The most common causes of erythroderma are eczema (40%), psoriasis (15%), drugs, and malignancies. Crusted scabies, lichen planus, and dermatomyositis account for only 0.5% of cases.^1,3^

## CASE REPORT

**Figure 1:**
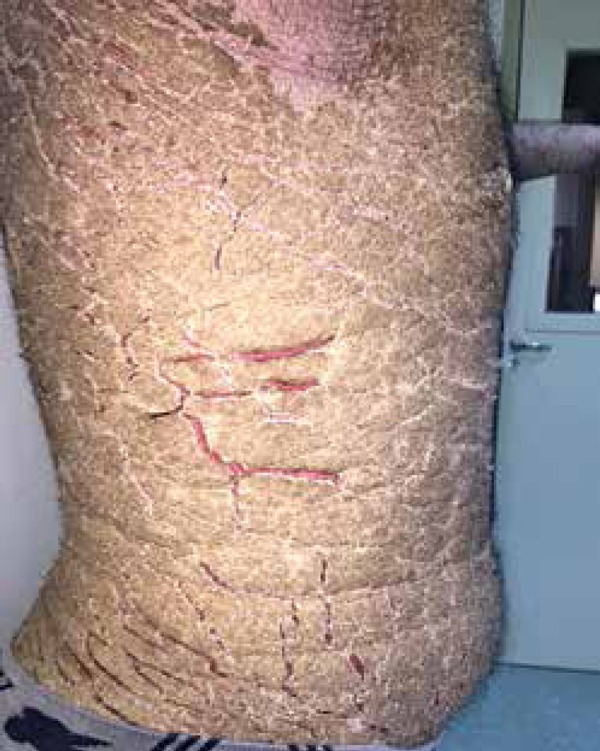
Anterior and lateral regions of trunk and abdomen with widespread crusted lesions

**Figure 2:**
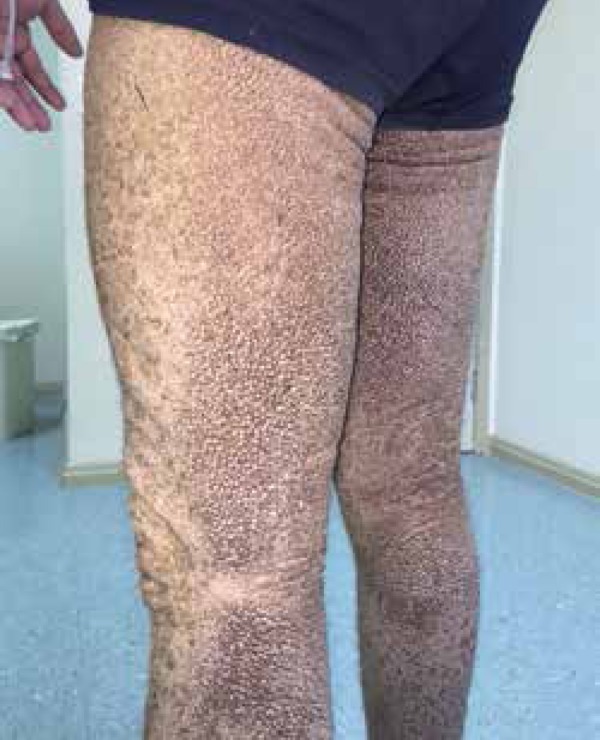
Lower limbs with widespread crusted lesions

**Figure 3:**
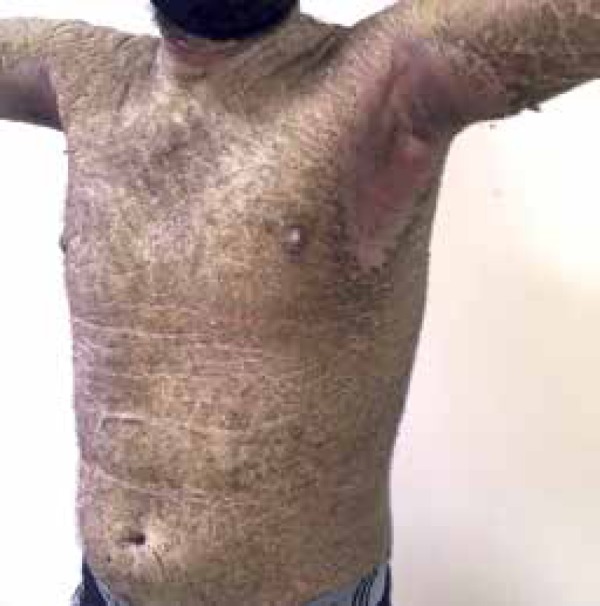
Anterior trunk region and abdomen with widespread lesions

**Figure 4:**
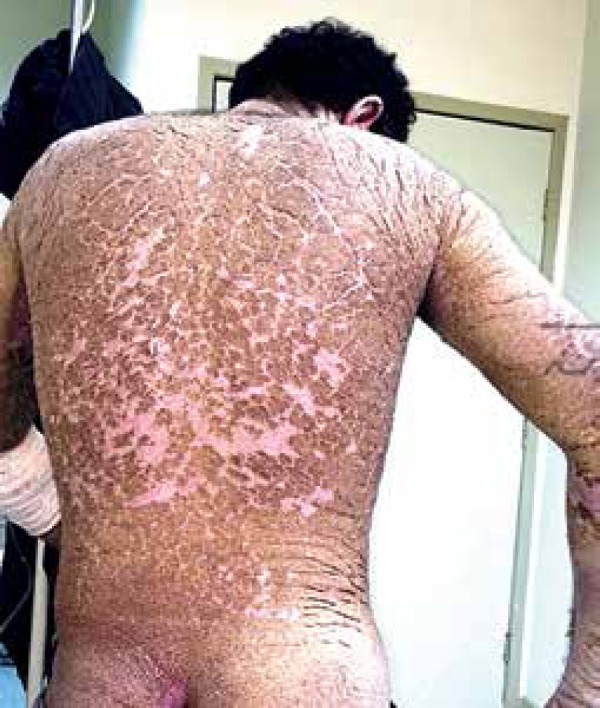
Back and buttocks with widespread lesions

This study reports the case of a patient whose clinical findings, after positive histopathological examination, allowed a quick diagnosis, reinforcing the increasing role that this technique has had in clinical practice. A 19-year-old male patient, born in Paraná, Brazil, began to show skin desquamation on the back of his hands and feet approximately 4 years before the consultation. The patient reported that, since then, the lesions had been gradually increasing until the penultimate month, when an outbreak of lesions spread throughout his body, except the palmoplantar regions (Figures 1 to 4). The lesions were non-pruritic, which hampered the initial diagnostic suspicion. No associated comorbidities were found, and the patient was not on any routine medication.

**Figure 5:**
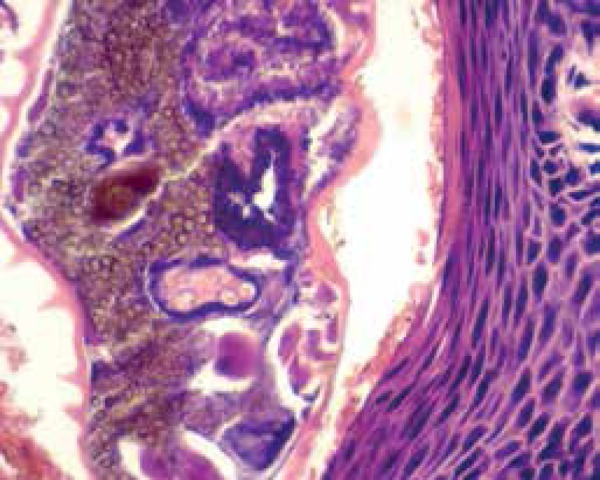
Skin. Epidermis with psoriasiform hyperplasia and several mites (Sarcoptes scabiei) in the stratum corneum (40x)

The patient was admitted to the emergency care unit with fever (38°C) without itching, pain, or other clinical manifestations. Therapy was initiated with intravenous corticosteroids, which worsened the crusted lesions. A week after the admission, a biopsy of the upper right dorsum was done; pathological examination reported crusted scabies as the final diagnosis (Figure 5). The patient also had an abscess on the gluteal region, which was surgically drained on the eighth day of admission. Sepsis was not observed.

**Figure 6:**
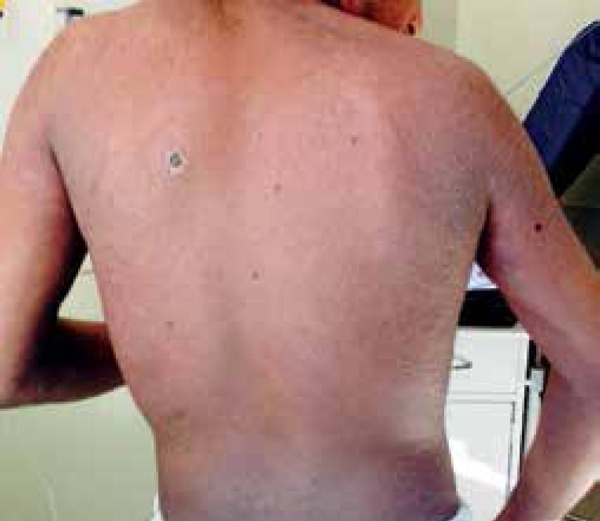
Back of the patient after 14 days of treatment

He was tested for HIV and HTLV-1, and serology for viral hepatitis and syphilis was performed. All tests and blood cultures were negative. The patient was transferred to the referral hospital and started treatment with four doses of ivermectin (18 mg), topical deltamethrin solution, and antihistamines in quarantine. He was also administered empirical doses of antibiotics due to the concomitant skin infection: amoxicillin + clavulanate and clindamycin (D4). Later, as the culture of soft tissues were consistent with *Enterococcus faecalis* sensitive to vancomycin, teicoplanin, and linezolid, the patient was treated with cefepime (D10) and vancomycin (D5). After 14 days of treatment, the lesions showed signs of regression, and itching and local pain improved significantly (Figure 6). All persons in contact with the patient, as well as the whole medical and nursing staff, were treated with ivermectin (oral), given the high risk of contagion. The patient was discharged after four weeks of admission, with almost complete regression of lesions.

## DISCUSSION

The association between scabies and neurologic deficit is common. However, the patient in this report was free from all kinds of cognitive, sensory, and motor impairment.^2^ Serology for HIV and cytomegalovirus were negative. In scabies, the host immune response is weakened, facilitating the intense multiplication of mites. However, this case is unusual because of the absence of concomitant immunosuppressive diseases.^1,2,4,5^

Due to differential diagnoses for hyperkeratotic eczema, psoriasis, and contact dermatitis, the authors opted for a corticosteroid therapy, which further aggravated the erythroderma, but with the unusual absence of associated pruritus.^4,6^ This symptom affected the diagnosis of scabies. However, the patient later reported that he had overlooked personal hygiene for six months, due to the pain and burning sensation during this procedure. This fact possibly masked the clinical presentation of the disease, raising doubts about its real etiology.

Some non-immunosuppressive risk factors such as neuropathy, severe arthropathy, cognitive deficit, and psychiatric disorders, incapacitate the patient to scratch the lesions.^7,8^ Scratching is important because it destroys the burrows of the mites, which did not occur with the patient in this case report.

Treatment for crusted scabies is difficult in some cases because of the large number of mites, forming hyperkeratotic areas in the skin. Therefore, a prolonged treatment with systemic scabicides is recommended, paying special attention to occacional concomitant infections.^4,5,8^ The most common treatment for crusted scabies is a single or recurrent dose of ivermectin (oral), depending on the severity of the case, as with the patient in this case.^2^ However, some pesticides and scabicides such as lindane might be applied.^7^ Pruridus and focal symptoms can also be treated with deltamethrin and 10% topical precipitated sulfur in petrolatum, for example.^4^

Another significant procedure is differential diagnosis with other dermatoses, such as crusted psoriasis - a rare form of the disease manifested by erythematous plaques covered with scabs,^3,8^ similar to the patient in this case.

## References

[1] Das A, Bar C, Patra A (2015). Norwegian scabies Rare cause of erythroderma. Indian Dermatol Online J.

[2] Guldbakke KK, Khachemoune A (2006). Crusted scabies a clinical review. J Drugs Dermatol.

[3] Burns DA, Burns T, Breathnach S, Cox N, Griffiths C (2010). Diseases caused by arthropods and other noxious
animals. Rook's Textbook of Dermatology.

[4] Towersey L, Cunha MX, Feldman CA, Castro CG, Berger TG (2010). Dermoscopy of Norwegian scabies in a patient with acquired
immunodeficiency syndrome. An Bras Dermatol.

[5] Berth-Jones J, Burns T, Breathnach S, Cox N, Griffiths C (2010). Eczema, lichenification, prurigo and erythroderma. Rook's Textbook of Dermatology.

[6] Johnston G, Sladden M (2005). Scabies diagnosis and treatment. BMJ.

[7] Cabral R, Coutinho I, Reis JP (2013). Caso para Diagnóstico - Escabiose humana. An. Bras. Dermatol.

[8] Costa JB, Rocha de Sousa VL, da Trindade Neto PB, Paulo Filho A T de, Cabral VC, Pinheiro PM (2012). Norwegian scabies mimicking rupioid psoriasis. An Bras Dermatol.

